# Universal Testing Apparatus Implementing Various Repetitive Mechanical Deformations to Evaluate the Reliability of Flexible Electronic Devices

**DOI:** 10.3390/mi9100492

**Published:** 2018-09-25

**Authors:** Cheol Kim, Chung Hwan Kim

**Affiliations:** 1 Department of Mechanical Engineering, Chungnam National University, 99 Daehak-ro, Daejeon 34134, Korea; kimchell@naver.com; 2 Department of Mechanical & Metallurgical Engineering Education, Chungnam National University, 99 Daehak-ro, Daejeon 34134, Korea

**Keywords:** flexible electronics, printed electronics, mechanical deformation, test apparatus, reliability

## Abstract

A requirement of flexible electronic devices is that they maintain their electrical performance during and after repetitive mechanical deformation. Accordingly, in this study, a universal test apparatus is developed for in-situ electrical conductivity measurements for flexible electrodes that are capable of applying various mechanical deformations such as bending, twisting, shearing, sliding, stretching, and complex modes consisting of two simultaneous deformations. A novel method of deforming the specimen in an arc to induce uniform bending stress in single and alternating directions is also proposed with a mathematically derived control method. As an example of the arc bending method, the changes in the resistance of the printed radio frequency identification (RFID) tag antennas were measured by applying repetitive inner bending, outer bending, and alternating inner-outer bending. After 5000 cycles, the increases in resistance of the specimens that were subjected to inner or outer bending only were under 30%; however, specimens that were subjected to alternating inner-outer bending showed an increase of 135% in resistance. It is critical that the reliability of flexible electronic devices under various mechanical deformations be determined before they can be commercialized. The proposed testing apparatus can readily provide various deformations that will be useful to inform the design of device shapes and structures to accommodate deformations during use.

## 1. Introduction

Flexible electronic devices that are bendable, foldable, or rollable are considered as next-generation electronics [1,2]. Example devices include flexible displays, flexible printed circuit boards, flexible touch screen panels, flexible solar cells, and flexible radio frequency identification (RFID) tag antennas [3,4,5,6]. These devices usually undergo mechanical deformations when they are used, however, they should maintain electrical performance during and after these deformations to guarantee reliability. Repetitive mechanical deformation is frequently observed in flexible electronic products such as wearable devices and foldable displays, and they can cause various failures such as cracking and delamination because of the resulting mechanical stresses [7]. Failures that are caused by fatigue have adverse effects on electrical performance and, therefore, fatigue tests should be conducted by observing the changes in the electrical performance of the device as it is used to ensure electrical reliability. In contrast to a conventional material test in which a thick bulk-metal sample is used as a specimen, the specimen in a flexible electronics material test is very thin. Therefore, the thickness effect should be considered and the range and methods of force and deformation application should be adjusted accordingly [8,9]. Moreover, the actual deformations that are experienced by flexible electronics when they are used commercially should be considered. Therefore, in addition to the representative research [10,11,12], there has also been a great deal of research related to the stability testing, material development, and design of thin flexible and stretchable devices: Both mechanical and electrical durability tests are usually conducted to ensure the reliability of the electrodes [3,4,13,14]; the effects of cyclic bending and/or tension on organic solar cells have been examined [15,16]; various materials that are suitable for flexible and stretchable devices have been developed [16,17,18]; and basic design methods for flexible device structure that are intended to enhance bending resistance have been proposed [19,20,21,22]. The final assessment step when determining the possibility of device commercialization is to perform a reliability test under repetitive mechanical loading. Therefore, a test method and apparatus that can accommodate such thin flexible electronic devices and their possible deformations is required.

**Figure 1:**
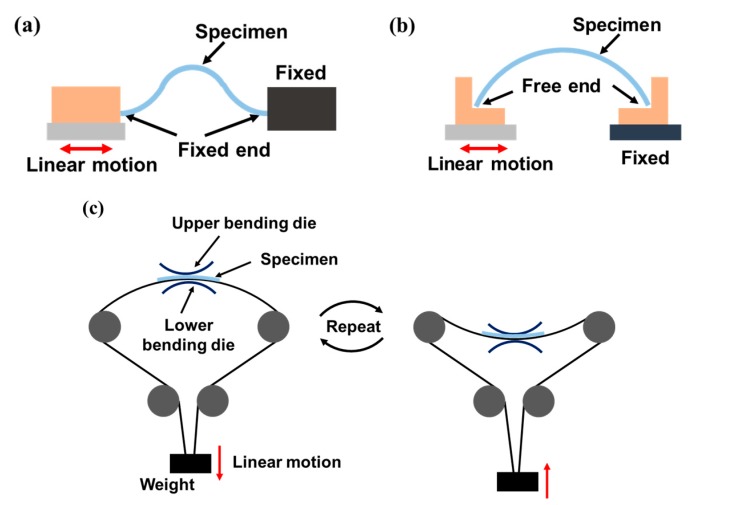
Schematics of conventional bending test methods for flexible electronic devices: (**a**) linear bending test with clamped ends; (**b**) linear bending test with free ends; (**c**) two-sided bending test using contact dies.

The fatigue life of flexible devices is predicted by measuring the change in electrical performance while applying repetitive mechanical deformation. In most previous studies, bending was applied to test the electrical reliability of a device using one of the representative methods that are illustrated in Figure 1a–c. Figure 1a is a schematic of the linear bending test method in which one clamp fixes one end of the flexible device and the other moves linearly [23]. In this configuration, the bending deformation and, consequently, the bending stresses are concentrated not only at the center of the specimen, but also at the ends near the clamps. These uneven and concentrated stresses can produce incorrect results if part of the specimen near the clamps fails first. Figure 1b also shows a bending test method using linear motion at one end, however because both ends of the specimen are free without clamps, the bending deformation and stress are relatively uniformly distributed [22,24,25,26]. However, in this clamp-free arrangement, it is difficult to measure the electrical properties of the specimen in-situ. Moreover, the bending direction is fixed in one direction, therefore, the tested device only undergoes compression (inner bending) and tension (outer bending) on one side. Figure 1c shows the schematic of a two-sided bending test method in which the direction of bending can be changed using dies, thus providing repetitive tension and compression stresses [27,28]. However, the minimum bending radius of the specimen is limited to that of the die and direct contact between the die and the specimen can cause additional unwanted contact stress, which can affect the electrical performance of the device in addition to the effects of a pure bending load. 

A fatigue test that applies twisting or stretching in addition to bending has also been developed. The simple method used to apply twisting is to rotate one clamp [26] or both clamps that fix the end of the specimen [29,30]. The general stretching test is implemented by moving one clamp [31] or both clamps in opposite directions [3,4,32] to extend the specimen.

In response to the growth in flexible device application fields and their consequent exposure to various mechanical deformations, various types of mechanical tests are required. A conventional test apparatus can generally provide only one test method, therefore, as many test apparatuses as test methods are required to test devices under various deformations. Moreover, a test apparatus that provides only a single test method cannot induce complex deformations such as bending plus twisting. In this study, a universal test apparatus that can provide various test methods of bending, sliding, twisting, shearing, and stretching was developed. In addition to the conventional bending method, a novel method is proposed that uses a mathematically derived model to inform a control algorithm, in which the specimen is subjected to alternating inner-outer bending in the shape of an arc to induce uniform stress along its entire length. This apparatus provides two types of complex deformation: bending and twisting, and bending and stretching. The apparatus can measure the electrical resistance of the specimen in-situ or off-machine using either the two-wire or four-wire method. As a test application, bending deformations due to compression (inner bending), tension (outer bending), and alternating compression-tension (inner-outer bending) were applied to printed RFID tags and the changes in their resistances were measured in-situ.

## 2. Development of Universal Test Apparatus

**Figure 2:**
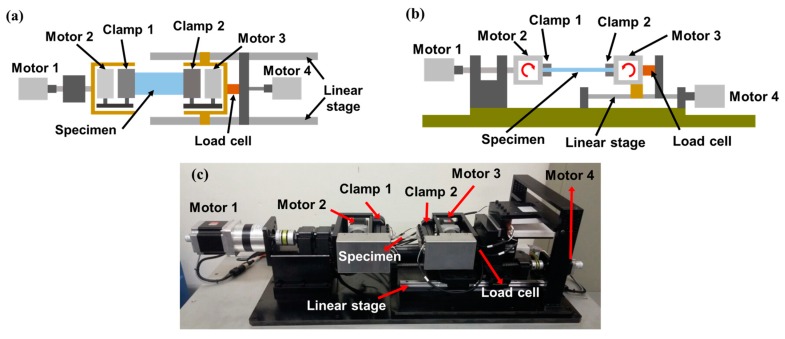
Developed universal test apparatus (**a**) schematic top view; (**b**) schematic side view; (**c**) actual photograph.

The test apparatus is divided into three components: a mechanical component that implements the deformation, consisting of four stepping motors, a linear stage, and a ball screw; a measurement component, including a resistance measurement device and a load cell for the real-time measurement of tension in the specimen; and a control component, which is based on the TwinCAT3 system that performs signal processing and motion control of motors, enabling the adjustment of the magnitude and frequency of the cyclic loading. Figure 2a,b illustrates schematics of the test apparatus, and Figure 2c is an actual photograph. The clamps play the role of fixing the specimen and also connect the electrodes of the specimen to the wires to measure specimen resistance. Figure 2 shows the basic configuration for applying bending, twisting, and stretching: the conventional linear bending deformation shown in Figure 1a can be implemented by the repetitive linear motion of Clamp 2 connected to Motor 4; stretching (tensile) deformation can be implemented by the linear motion of Clamp 2 by Motor 4 as well, and can be measured using the load cell; twisting deformation can be induced by the rotational motion of Clamp 1 with the help of Motor 1. Motors 2 and 3, which rotate Clamps 1 and 2, respectively, are used to implement the proposed novel bending mode, which will be described in detail in Section 3.

**Figure 3:**
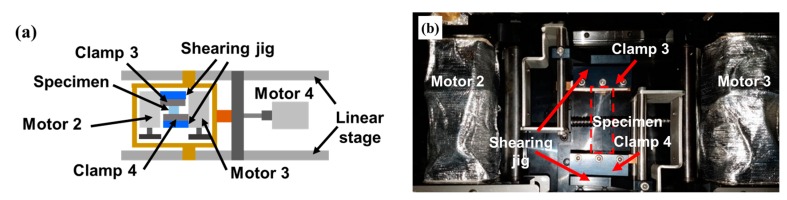
Configuration for shearing test (**a**) schematic; (**b**) actual photograph.

**Figure 4:**
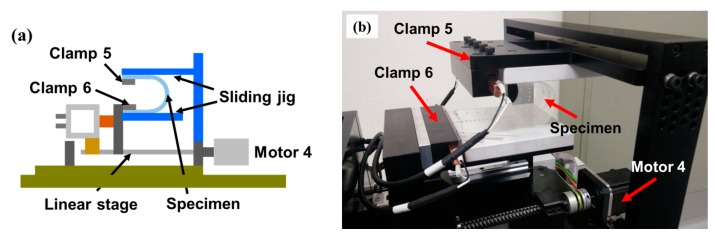
Configuration for sliding test (**a**) schematic; (**b**) actual photograph.

Figure 3 shows the top view of the configuration of the developed test apparatus for a shearing test in which a jig is added to change the direction of the specimen so that it is perpendicular to the linear motion that is applied by Motor 4. One end of the specimen (at Clamp 4) is connected to Motor 4, and its linear motion applies shearing deformation to the specimen. Figure 4 shows the configuration for a sliding test [7,33,34] in which a jig is added so that the repetitive linear motion of Clamp 6 which is connected to Motor 4 applies a repetitive sliding motion to the specimen. 

Because the resistance measurement unit has four wires, the resistances of two different electrodes can be simultaneously measured using the two-wire method by using two of the wires as common grounds and the other two wires as separate connectors. This arrangement enables resistance measurements of two different types of electrodes simultaneously. This configuration is also very useful when conducting a twisting test, as the magnitude of twisting is dependent on the distance of the specimen from the twisting axis. This allows for different twisting effects to be applied to different devices of the same type when they are simultaneously installed at different positions relative to the twisting axis. For example, one measurement can be conducted with the device oriented along the twisting axis, while another can be simultaneously conducted with the device offset from the axis, allowing for the determination of the effect of twist magnitude on the device. 

## 3. Application of Mechanical Deformations 

### 3.1. Application of Conventional Mechanical Deformations 

**Figure 5:**
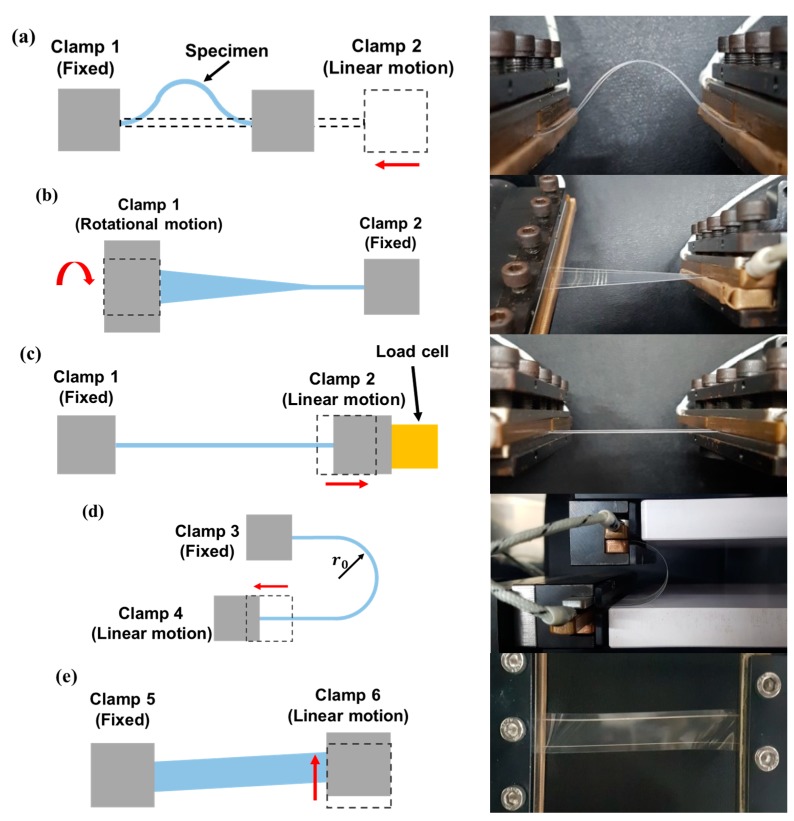
Schematics and actual photographs of various deformation modes that are implemented by the proposed test apparatus: (**a**) linear bending mode; (**b**) twisting mode; (**c**) stretching mode; (**d**) sliding mode; (**e**) shearing mode.

Figure 5a–d show the schematics of methods for applying mechanical deformations that have been proposed in previous works using the developed test apparatus. Figure 5a shows a schematic as well as an actual photograph of the apparatus in the linear bending test mode. As Clamp 2, which is connected to Motor 4, moves linearly toward the fixed Clamp 1, the specimen undergoes bending. However, as described in Section 1, additional bending occurs near the clamps. Figure 5b shows a schematic as well as an actual photograph of the apparatus in the twisting mode [26]. Because of the rotating motion of Clamp 1 connected to Motor 1, the specimen is subjected to a torsional load, and the magnitude of twist (twisting angle) is controlled by Motor 1. Figure 5c shows a schematic as well as an actual photograph of the apparatus in stretching mode, in which Clamp 2 moves away from Clamp 1 to induce tension in the specimen [31]. The tension is measured by the load cell and is controlled by the control system. Figure 5d shows a schematic and an actual photograph of the apparatus in sliding mode [7,33,34], in which the specimen is fixed with an initial curvature of $r_{0}$ and then deforms as Clamp 4, which is connected to Motor 4, moves linearly. The radius of curvature is constant during this deformation. Figure 5e shows a schematic and an actual photograph of the apparatus in shearing test mode, which has not been addressed in other work, however it is possible with the proposed apparatus. In this case, Clamp 5 is fixed, however Clamp 6 moves perpendicular to the length of the specimen, inducing shear in the specimen. 

### 3.2. Application of Novel Bending Deformation

As described in Section 1, all conventional bending tests have drawbacks: non-uniform bending stress (Figure 1a); limited bending direction (Figure 1b); and uniform bending stress under alternating bending direction, but limited bending radius and possible contact stress (Figure 1c). In this section, a bending method that addresses the drawbacks of these conventional methods is proposed: an alternating bending deformation that can exert uniform bending stress on a specimen. 

#### Mathematical Model for Bending a Specimen into an Arc Shape

To apply a uniform bending moment to a specimen by deforming it into an arc, a mathematical model for calculating the motor control parameters using the geometric relationship between arc geometry and motor rotation is derived. The bending moment in the specimen is expressed as [35]:

(1) $$M(x)=-E(x)I(x)\frac{y^{\prime \prime }}{\sqrt{{\left[1+{\left(y^{\prime }\right)}^{2}\right]}^{3}}}\;$$

where E, I, and y are the Young’s modulus, moment of inertia, and deflection, respectively, of the specimen. Because the induced arc is part of a circle, variables $y^{\prime }$ and $y^{\prime \prime }$ can be obtained using the circle equation,

(2) $$x^{2}+y^{2}=r^{2}\;$$

The variables can then be expressed as:

(3) $$y^{\prime }=\frac{dy}{dx}=-\frac{x}{y}\;$$

(4) $$y^{\prime \prime }=\frac{d^{2}y}{dx^{2}}=-\left(\frac{x^{2}+y^{2}}{y^{3}}\right)\;$$

By substituting Equations (3) and (4) into Equation (1), the bending moment of the arc-shaped deformation can be expressed by assuming a constant Young’s modulus and cross section as:

(5) $$M(x)=\frac{E(x)I(x)}{r}=\frac{EI}{r}\;$$

**Figure 6:**
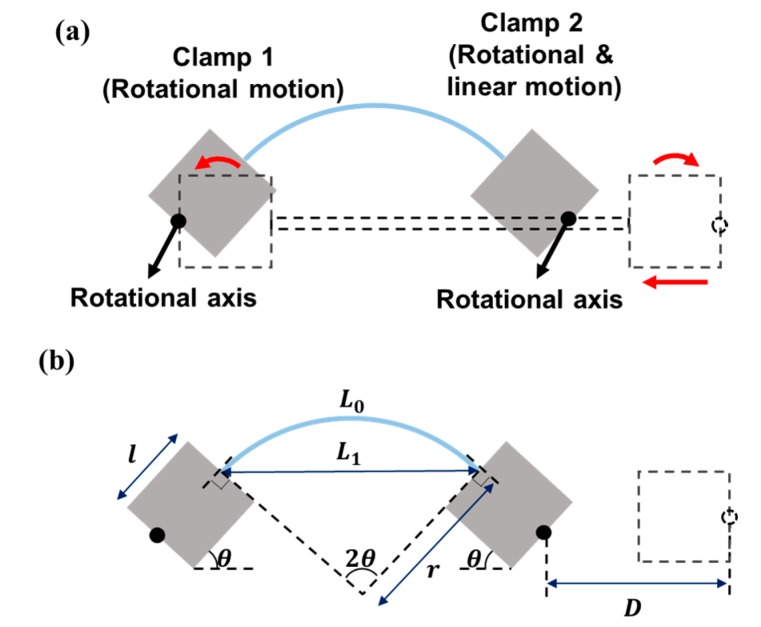
(**a**) Schematic of the deformed specimen in an arc shape; (**b**) related variables and geometric properties.

As shown in Figure 6a, the specimen is deformed into an arc by the motions of two clamps that are connected to three motors of Figure 2: Clamp 1 undergoes rotational motion by means of Motor 2, while Clamp 2 undergoes rotational and linear motion by means of Motors 3 and 4, respectively. Figure 6b shows the variables that describe the geometric properties before and after the specimen is deformed into an arc. As the initial horizontal distance $L_{0}$ between the two clamps decreases to $L_{1}$, the specimen deforms into an arc with a radius of r. Because the strain in the specimen during deformation is very small, we can assume that the length of the specimen after deformation is identical to its initial length $L_{0}$. The clamps should rotate by θ so that the arc is perpendicular to both clamps in order to prevent the specimen from developing stress concentrations at the clamping points. Note that 2θ corresponds to the central angle of the arc. The relationship between horizontal distances $L_{0}$ and $L_{1}$ is written as

(6) $$L_{1}=L_{0}-D+2l(1-\cos\theta ),$$

where l and D are the length of the clamp and the moving distance of the rotational axis of Clamp 2, respectively, in the horizontal direction. The values of $L_{0}$ and $L_{1}$ are expressed in terms of the radius of curvature r and the rotation angle of the clamp θ as:

(7) $$L_{0}=2r\theta \;$$

(8) $$L_{1}=2r\sin\theta \;$$

By combining Equations (6)–(8), the relationship between the rotational angle θ and the moving distance of the clamps D is expressed as:

(9) $$D=L_{0}+2l(1-\cos\theta )-\frac{L_{0}}{\theta }\sin\theta \;$$

**Figure 7:**
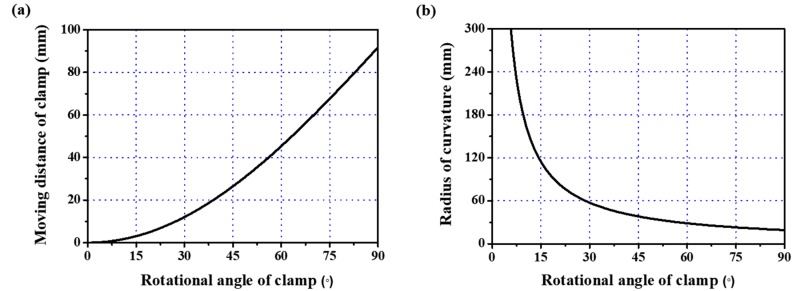
Calculation results of parameters to obtain the desired radius of curvature of the bent specimen: (**a**) relationship between the rotational angle and moving distance of Clamp 2; (**b**) relationship between the rotational angle and radius of curvature.

**Figure 8:**
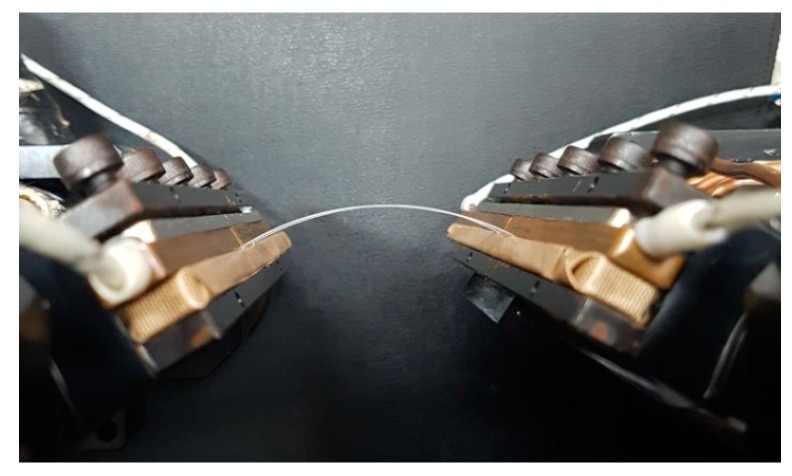
Deformed specimen in an arc shape.

It is easy to calculate D given a value of θ. Therefore, the moving distance of Clamp 2, or D, and the rotational angle of Clamps 1 and 2, or θ, that are required to deform the specimen to the desired radius of curvature r can be obtained from Equation (9). Figure 7a,b shows the calculated moving distance and the radius of curvature, respectively, as the rotational angle linearly increases, for $L_{0}$ = 60 mm and l = 35 mm. As the rotational angle increases, the moving distance increases and the radius of curvature decreases. If we select the radius of curvature of the specimen in Figure 7b, the required moving distance and rotational angle of the clamps, as implemented in the control system, can be calculated from Figure 7a. Figure 8 shows the arc shape of the deformed specimen based on the proposed mathematically derived control method. The bending method that is shown in Figure 6 and Figure 7 is suitable for applying uniform bending stress to large area devices ranging from several tens of millimeters in length. Therefore, a very small curvature in the sub-millimeter range is not possible with this bending method. However, a small radius of curvature can be realized in two ways: first, when using the bending method shown in Figure 5a, which is also a possible configuration in the proposed apparatus, the small device can be located at the center of the specimen; second, because we can control the rotational angle and linear movement of each clamp independently, concentrated bending at the center is also possible by modifying the proposed rotational bending method so that the flexible device is connected to two separate, comparably rigid plates that are themselves connected to the clamps, such that the application of a larger rotational angle θ causes both of the plates and the flexible device specimen to deform in a reversed ‘V’ shape, thus creating concentrated bending with a very small radius of curvature.

### 3.3. Application of Complex Deformations

**Figure 9:**
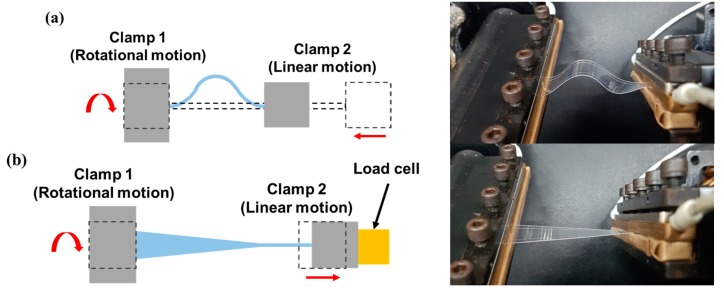
Schematics and actual photographs of complex deformation modes: (**a**) twisting and bending; (**b**) twisting and stretching.

Flexible devices can simultaneously experience more than one type of deformation when they are used. For example, a skin patch with sensor arrays to monitor body conditions, which is one of the target applications of wearable devices, can undergo both bending and twisting at the same time when attached to the skin. In this section, examples of complex deformations in which a specimen undergoes two simultaneous deformations are proposed. Figure 9a shows a schematic and an actual photograph of combined twisting and bending, in which Clamp 1 applies a rotational motion with Motor 1, while Clamp 2 applies linear motion using Motor 4. Figure 9b depicts combined twisting and stretching, in which Clamp 1 applies rotational motion using Motor 1, while Clamp 2 applies tension to the specimen by pulling with Motor 4 and measures it with the load cell.

As described in Section 3 and Section 4, the characteristics of the developed test apparatus and its advantages over conventional apparatus can be summarized as follows: (1) the developed apparatus is capable of various types of repetitive mechanical loading (linear bending, rotational bending including the proposed bending method, concentrated bending, twisting, stretching, combined sliding and shearing, combined twisting and linear bending, and combined twisting and stretching); (2) time scheduling of different loading types to conduct an application or usage scenario test is possible; (3) the magnitude and frequency of the cyclic loading can be adjusted; (4) resistance can be measured in-situ or off-machine; and (5) the resistance of two samples can be measured simultaneously.

Note that because all of the motors in the proposed apparatus are controlled by the control system, and because the clamps that are connected to the motors can provide independent rotational and linear motion, various configurations to provide deformations other than those discussed in this paper can be implemented as well.

## 4. Evaluation of the Electrical Reliability of Flexible Printed RFID Tag Antennas under Repetitive Bending Deformation

**Figure 10:**
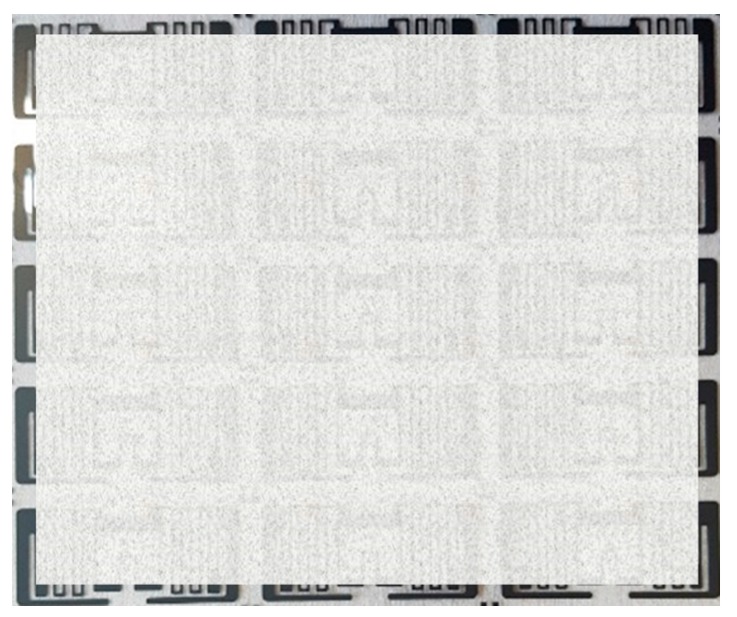
Photographic image of the specimens that were used for repetitive bending tests (A significant portion of the photograph is obscured to protect the confidentiality of the RFID Company’s antenna design).

As an example application of the developed apparatus to evaluate the electrical reliability of flexible devices, the arc-shaped bending deformation that was proposed in Section 3.2 was applied to a flexible printed device. To serve as specimens, RFID tag antennas were fabricated on polyethylene terephthalate (PET) by the screen printing method using conductive silver nanoparticle ink. Figure 10 shows a photograph of a sheet of printed RFID tag antenna arrays. Note that a significant portion of the photograph is obscured to protect the confidentiality of the RFID company’s antenna design. The two ends of the antenna were clamped and were connected to wires to monitor its resistance. While applying the mechanical deformation, the resistance of the printed antenna was acquired in-situ by the two-wire resistance measurement unit which was contained by the proposed apparatus.

**Figure 11:**
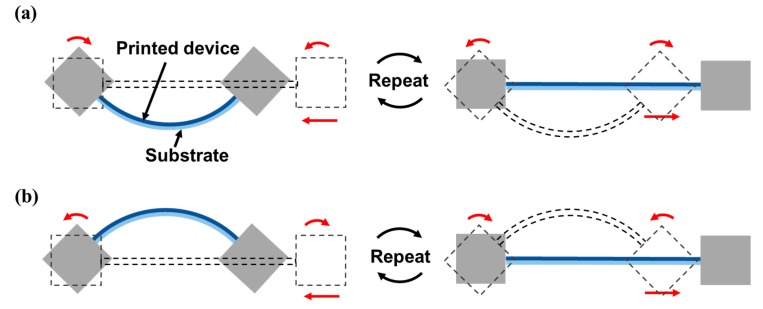

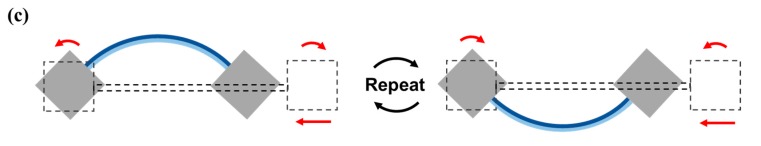
Schematics of printed antenna bending modes under different loadings: (**a**) compressive stress by one-sided bending (inner bending); (**b**) tensile stress by one-sided bending (outer bending); (**c**) alternating tensile and compressive stresses by two-sided bending (inner-outer bending).

As shown in Figure 11a–c, two types of bending modes were repetitively applied. Figure 11a,b depict the same one-sided bending mode, but with the printed antennas on opposite sides. In Figure 11a, the antenna is located on the inner side of the arc and, therefore, the antenna only experiences compressive stress (inner bending). In Figure 11b, the antenna is located on the outer side of the arc, therefore, the antenna only experiences tensile stress (outer bending). Figure 11c depicts the two-sided bending mode that deforms the specimen in alternate directions, in which the printed antenna is alternately subjected to tensile and compressive stress, regardless of the location of the printed antenna (inner-outer bending). 

The specimens had initial resistances of 5 ± 0.3 Ω and their resistances were measured regularly when the deformations were at their minimum and maximum. All of the experiments were conducted at room temperature under the detailed experimental conditions that are listed in Table 1. 

**Figure 12:**
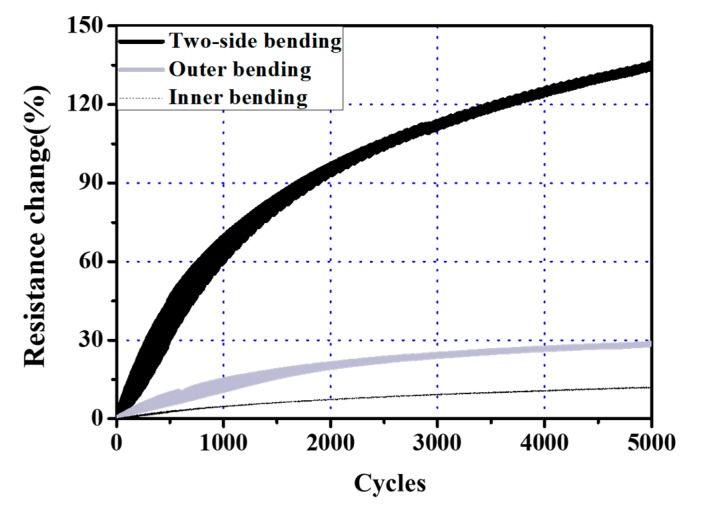
Change in resistance of RFID sample under three different bending loads.

The measured resistances of the antennas were transformed into resistance change using Equation (10):

(10) $$R_{1}=\left(\frac{R-R_{0}}{R_{0}}\right)\times 100\%,$$

where $R_{0}$, R, and $R_{1}$ are the initial resistance, measured resistance, and resistance change, respectively. Figure 12 shows the resistance change of the printed pattern under the three different bending load regimes. As can be seen in this figure, the three cases show different rates of resistance change. After 5000 cycles, the resistances of the antennas under one-sided compressive and tensile bending were 12% and 29% higher, respectively. On the other hand, in the case of two-sided bending, the resistance was 135% higher than its initial value, or 11 times higher than the value that was reported after the one-sided bending with compressive force. One-sided bending deformation under compressive load results in a smaller change in resistance than under tensile load, and this difference can be explained by reference to similar experimental results in previous research [24,34,36]: local cracks or delamination may occur in a specimen under repetitive compressive load, however the change in resistance is smaller than that under tensile load because of slow crack propagation [36]. In the case of two-sided bending, crack propagation is accelerated and, consequently, the resistance increases more rapidly with increasing cycles than in the cases of only inner or outer bending. Therefore, in order to ensure the electrical reliability of commercial flexible devices, it is necessary to conduct mechanical tests using two-sided bending. The proposed apparatus can fulfill this need quite effectively.

## 5. Conclusions

A universal test apparatus was proposed for the reliability testing of flexible electronic devices that can apply various mechanical deformation modes such as bending, twisting, shearing, sliding, and stretching, as well as complex modes in which two deformations are implemented at the same time. The developed apparatus is equipped with an in-situ resistance measurement unit to measure the changes in the specimen resistance and a load cell to measure the tension in the specimen. In addition to the conventional bending test methods, a novel test in which the specimen is deformed into an arc with its ends perpendicular to the clamps, such that it undergoes fully uniform bending stress, was proposed based on a mathematically derived control method. The proposed bending method can provide a two-sided (inner-outer) bending testing in which the specimen undergoes alternating compressive and tensile forces. The developed universal test apparatus can be used to predict the fatigue life and failure mode of flexible electronic devices by accurately reproducing the characteristics of the mechanical deformations that they experience when they are used in their intended applications. The effects of these various deformations on a device can also be analyzed to determine the types of deformations that are having the most serious effect. By gathering data from reliability tests under various mechanical deformations, the shapes and structures of devices can be designed to endure or to reduce the effects of the most damaging types of mechanical deformation. Accordingly, the developed apparatus can contribute to an improvement in the reliability of flexible electronics and could accelerate their commercialization.

As an application example, the changes in the resistances of printed RFID tag antennas were measured by applying repetitive inner bending, outer bending, and alternating inner-outer bending. After 5000 cycles, the increases in resistance that were due to inner only and outer only bending were 12% and 29%, respectively, while those that were due to alternating inner-outer bending were as high as 135%. This result implies that the reliability of flexible electronic devices should be evaluated using two-sided bending tests in order to guarantee quality when they are used commercially as wearable and flexible electronic products.

In future work, reliability tests using complex modes and reliability tests of other flexible devices, such as organic light emitting diode displays or lighting, solar cells, and sensor arrays, under various mechanical deformations should be evaluated. 

## Figures and Tables

**Table 1:** Experimental conditions for rotational bending tests.

Parameters	Value
Initial length of the specimen ($L_{0}$, mm)	31.0
Rotational angle of the clamps (θ, rad)	π/6
Moving distance of Clamp 2 (D, mm)	12.1
Radius of curvature (r, mm)	29.6
Motion frequency (Hz)	0.4
